# Comparison of Flavor Differences between the Juices and Wines of Four Strawberry Cultivars Using Two-Dimensional Gas Chromatography-Time-of-Flight Mass Spectrometry and Sensory Evaluation

**DOI:** 10.3390/molecules29194691

**Published:** 2024-10-03

**Authors:** Wei Lan, Wei Cheng, Ruilong Li, Mei Zhang, Mengmeng Li, Yuan Zhang, Yibin Zhou

**Affiliations:** 1 School of Biology and Food Engineering, Fuyang Normal University, Fuyang 236037, China; lanwei@fynu.edu.cn (W.L.); 13805585071@163.com (W.C.); pennylrl@163.com (R.L.); 22211310@stu.fynu.edu.cn (M.Z.); limengmeng@fynu.edu.cn (M.L.); zy7704@foxmail.com (Y.Z.); 2 Anhui Engineering Research Center for Functional Fruit Drink and Ecological Fermentation, Fuyang 236037, China; 3 College of Food and Nutrition, Anhui Agricultural University, Hefei 230036, China

**Keywords:** strawberry juice, strawberry wine, volatile organic compounds, two-dimensional gas chromatography–time-of-flight mass spectrometry, sensory evaluation

## Abstract

Fruit wine production is a practical approach for extending the shelf life and enhancing the value of strawberries (*Fragaria* × *ananassa*). Fruit cultivars and juices are important sources of volatile organic compounds (VOCs) that determine fruit wine sensory quality. In this study, VOCs in the juices and wines of four strawberry cultivars were identified using two-dimensional gas chromatography-time-of-flight mass spectrometry, and a sensory analysis of the wines was performed. A total of 1028 VOCs were detected. PCA and OPLS-DA distinguished the four cultivars from which the juices and wines were made. Six VOCs with variable importance in projection values greater than one were the main aroma and flavor components of strawberry wines. ZJ wine had the highest sensory scores for coordination (9.0) and overall evaluation (8.9) among the 18 descriptors of strawberry wine evaluated. Overall, the ZJ wine had the highest alcohol content (13.25 ± 0.59%, *v*/*v*) and sensory evaluation score, indicating that the ZJ cultivar is more suitable for fermentation. This study reflects the differences between wines made from four strawberry cultivars and provides a reference for brewing fruit wines.

## 1. Introduction

Fruit wine, one of the oldest fermented beverages made from non-grape fruits, is generally produced by first extracting juice from the fruit and then brewing it through processes such as fermentation, soaking, distillation, or storage [1,2]. The quality of fruit wine is affected by many factors, with the quality of the raw materials being the main factor that directly affects the taste, flavor, and nutritional content of the product. The nutritional composition of fruits (sugars, organic acids, etc.) differs according to the fruit variety, growing region, climate, and harvest season [2,3,4]. Therefore, the fermentation process varies for particular fruits selected for wine production, resulting in content differences in volatile organic compounds (VOCs), which ultimately determine the sensory quality of the wine [3]. During the brewing process, the main factors affecting the quality of fruit wine include the ratio, initial sugar content, and inoculation amount of the raw material, as well as fermentation temperature, time, and pH [2,3,4,5]. Moreover, the optimal fermentation conditions for different raw fruit materials differ slightly [6,7,8]. Therefore, the development of fermentation techniques to enhance the VOCs and sensory attributes of fruit wines would help increase the market share of such wines, especially products with Chinese-favored characteristics.

Strawberry (*Fragaria* × *ananassa*) wine production is a practical approach to extend the shelf life and enhance the value of the fruit, with the fruit nutrients preserved through the fermentation process [9,10]. However, although the concentrated harvesting period and short shelf life of strawberries can be resolved by turning the fruits into wine, the appropriate cultivars to use for alcoholic fermentation remain unknown [6,11,12,13]. Although cultivar selection is a prerequisite for quality control of raw materials for fruit winemaking, knowledge regarding the fermentation quality of different strawberry cultivars remains limited. Moreover, aside from the basic characteristics that provide fruit wine with nutritional and health benefits, flavor components and their composition are also important for determining the quality and typical flavor of wine [14,15]. More than 360 volatile flavor compounds have been identified in strawberries and strawberry wine, and the aromas of different fruit cultivars are characterized by distinct combinations of compounds such as alcohols, esters, organic acids, aldehydes, ketones, terpenes, and sulfur compounds [15]. However, the differences in VOCs between strawberry juice and wines of different cultivars have not been fully elucidated, and the influence of juice on the VOCs of wine remains unknown.

VOC profiles of different fruit wines are typically studied using various pretreatment methods and detection equipment. Headspace solid-phase microextraction (HS-SPME), which has been widely used to study VOCs in fruit wine and other alcoholic beverages, is a simple and fully automatic operation with no complex pretreatment steps [15,16,17]. Moreover, two-dimensional gas chromatography (GC × GC) has a higher peak capacity, higher sensitivity, and better substance-separating power than those of its one-dimensional counterpart, and coupled with time-of-flight mass spectrometry (TOF-MS), GC × GC–TOF-MS has been successfully applied to detect thousands of compounds in distilled liquors or wines [17,18,19]. For example, HS-SPME coupled with GC × GC-TOF-MS was used to identify 196 biomarkers among 1695 compounds detected in Italian sparkling wines, thereby facilitating the determination of the influence of grape cultivars, pedoclimatic conditions, and metabolomic space on these wines [19].

In this study, we aimed to determine the VOC profiles of the juices and wines of four typical strawberry cultivars widely cultivated in China: Snow White (BX), Sweet Charlie (TCL), Tongzhougongzhu (TZ), and Zhangji (Akihime, ZJ). GC × GC–TOF-MS combined with HS-SPME was used to detect and identify VOCs. Sensory analysis of the four wines produced (designated as BX, TCL, TZ, and ZJ) was performed to distinguish the source and variety of strawberry raw materials for wine fermentation. Our results provide valuable information for the in-depth brewing of strawberry wine.

## 2. Results and Discussion

### 2.1. Sensory Evaluation of Strawberry Wines

**Figure 1:**
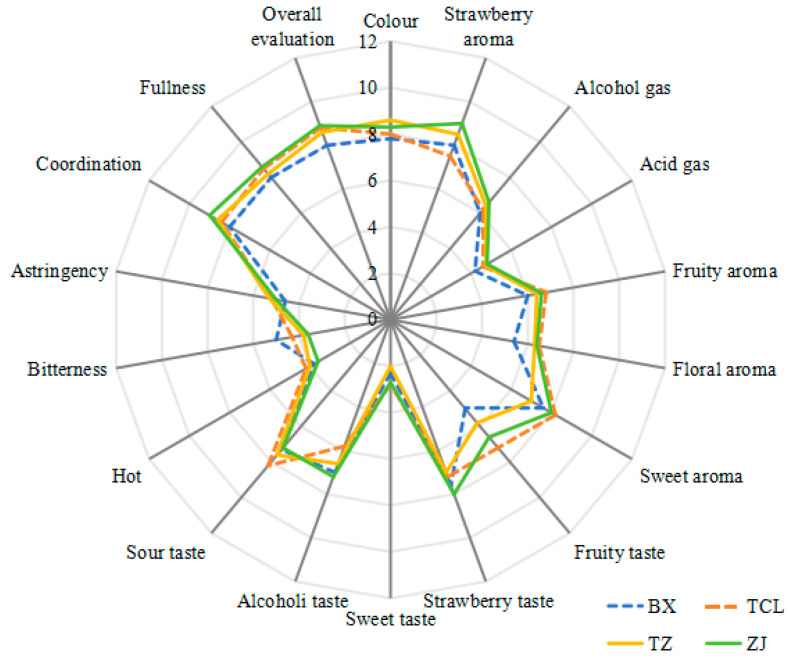
Sensory evaluation of four strawberry wines designated as BX, TCL, TZ, and ZJ. The 18 descriptors and their corresponding reference samples are shown. BX, Snow White; TCL, Sweet Charlie; TZ, Tongzhougongzhu; ZJ, Akihime.

In sensory evaluations, both volatile and non-volatile components are assessed simultaneously, and strawberry wine is mainly characterized by its appearance, aroma, taste, and overall body [11,15]. In this study, the sensory characteristics of four strawberry wines were quantitatively and descriptively analyzed, and 18 descriptors and their corresponding reference samples were identified (Figure 1). The sensory profiles of the four wines differed significantly based on fruit and flower notes. In terms of aroma attributes, TCL had the highest scores for fruity and floral notes, whereas ZJ had the highest scores for coordination (9.0) and overall evaluation (8.9). In contrast, BX had the lowest coordination and overall evaluation scores.

Floral aromas are associated with the presence of phenylethanol, geraniol, citronellol, and nonanal compounds, as well as the aromatic activities of ethyl acetate, ethyl propanoate, 3-methyl 1-butanol, methyl butanoate, and ethyl hexanoate [20]. In summary, the sensory profiles of strawberry wines are primarily influenced by their VOC composition.

In terms of taste, ZJ had the highest score for alcohol thickness, TCL had the highest score for sour and sweet palatability, and BX had low sour and sweet palatability scores and lacked alcohol thickness and persistence. In addition, ZJ had the highest scores for harmony, fullness, and overall evaluation. In general, the sensory analysis highlighted the different aromas and taste descriptors of the four strawberry wines, and six of the 18 sensory attributes (strawberry aroma, fruity aroma, sweet aroma, strawberry taste, sour taste, and bitterness) were emphasized in two or three samples. Therefore, these sensory attributes may be characteristic of strawberry varieties.

### 2.2. Physicochemical Properties of Juices and Wines

For fruit wines, ethanol and residual sugar contents are the main quality indicators of fermentation [2,11]; those of the fermented strawberry juices of the four cultivars are shown in Table 1. The tastes of fruit juices and wines were also affected by the pH and total acid content; those of the four samples are shown in Table 2. After fermentation, the ethanol content of the four strawberry cultivars was >13.00% (*v*/*v*) (Table 1), and the residual sugar content was <4.00 g/L (Table 1), which is the standard for dry fruit wines. The pH values of the non-fermented strawberry juices varied among the different cultivars, with BX juice (BX_J) having the highest value (4.06) and TCL_J having the lowest (3.36) (Table 2). With regard to wines, BX had the highest pH value (4.03), whereas TCL had the lowest pH (3.53), indicating that the fermentation process had little effect on pH. In alcoholic fermentation, pH is a critical parameter, as it affects the growth of yeasts and the characteristics (e.g., color, aroma, and taste) of the final alcohol product. For most types of fermentation, a pH range of 2.80–4.00 is considered ideal [21,22] and is suitable for the growth of yeasts and other brewing microbes.

The ZJ wine had the highest alcohol content (13.25 ± 0.59%, *v*/*v*) among the four wines, whereas its residual sugar content, pH value, and total acid content were at comparatively moderate levels among the different samples. These indicators may affect the harmony, fullness, and overall evaluation of wines. Additionally, differences in the pH, residual sugar content, and total acidity of strawberry juice resulted in significant differences in the physicochemical properties of the wines after fermentation. These indicators may affect substance metabolism during brewing and further influence the quality characteristics (e.g., aroma and taste) of wine [23].

The physicochemical properties of strawberry wines are closely related to the raw materials (cultivars, origins, and states), microbial strains, and fermentation technologies used, whereas other factors such as the color, antioxidative properties, and VOCs of strawberry wines fermented with juice have been found to differ from those of wines fermented with pulp [11,14,24]. Furthermore, aside from having a lighter color, strawberry wines fermented from the juice of pulp macerated at 50 °C have more desirable qualities and pleasant floral and fruity notes than those of wines fermented from the juice of pulp macerated at normal temperatures [24].

### 2.3. Identification and Statistical Analysis of Volatile Organic Compounds

#### 2.3.1. Identification of Volatile Organic Compounds

**Figure 2:**
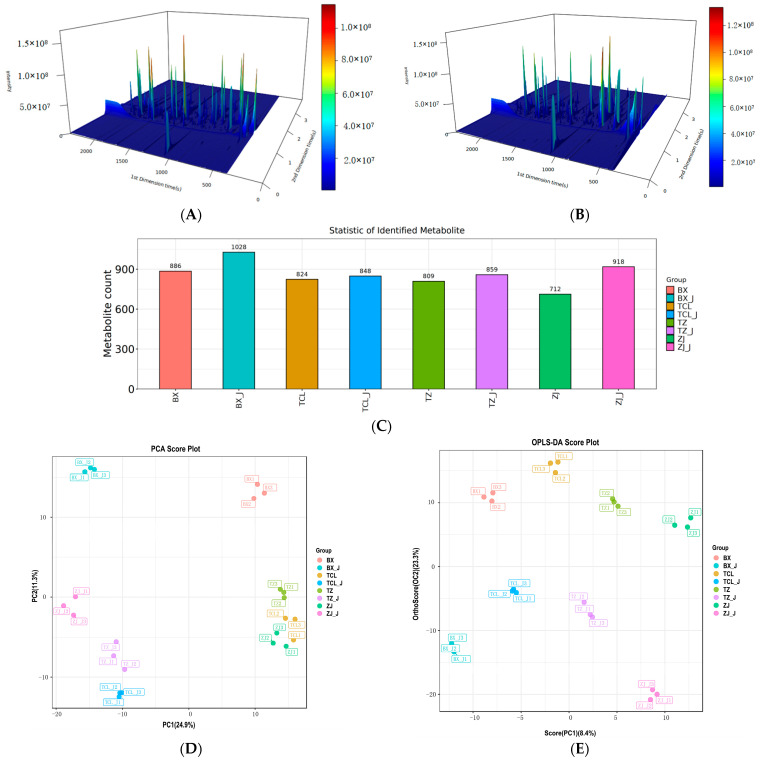
VOCs identified in four strawberry juices (_J) and wines. (**A**) Three-dimensional total ion chromatogram (TIC) of BX_J. (**B**) Three-dimensional total TIC of BX wine. (**C**) Quantitative comparison of VOCs. (**D**) PCA of VOCs. (**E**) OPLS-DA scores of VOCs.

VOCs determine the sensory characteristics of food and are of great utility in the food industry as markers of authenticity. Aroma plays a vital role in shaping flavor profiles and is a key attribute that affects consumer satisfaction [11]. The combined qualitative and quantitative analysis of VOCs using chromatography and MS techniques overcomes the shortcomings of separate traditional chromatography, MS, and qualitative data and provides the added benefits of simplified operation and shortened analysis time [25,26]. Compared to GC-MS, GC × GC-TOF-MS uses two columns with different properties to separate compounds that have not been completely separated in the first dimension, thereby facilitating the detection of a more comprehensive range and a higher quantity of volatile flavor compounds [17,25]. As shown in Figure 2, a good classification of VOCs in strawberry wines and juices was obtained (Figure 2A,B), and the number of VOCs identified was higher than that identified by GC-MS [25].

The quantities of VOCs detected in strawberry wines and juices by GC × GC-TOF-MS are shown in Table 3. In total, 886 metabolites were identified in the BX wine (Figure 2C), including 48 ketones, 88 hydrocarbons, 16 heterocyclic compounds, 9 aldehydes, 130 esters, 82 alcohols, 35 carboxylic acids, and 478 other compounds (Table 3). In contrast, 1028 substances were identified in BX_J (Figure 2C), including 63 ketones, 109 hydrocarbons, 26 heterocyclic compounds, 20 aldehydes, 123 esters, 96 alcohols, 39 carboxylic acids, and 552 other compounds (Table 3). Strawberry aroma is characterized by a combination of different compounds, each of which contributes to the characteristics of different cultivars [15]. In the present study, the VOC detection results indicated that the aroma of strawberry wine was characterized by a combination of ketones, hydrocarbons, heterocyclic compounds, aldehydes, esters, alcohols, and carboxylic acids.

Esters, which impart a characteristic aroma and flavor to strawberries, were the main VOCs detected in the four wines and juices, with hexanoic acid ethyl ester, heptanoic acid methyl ester, ethyl acetate, and methyl hexanoate being the most abundant (Table S3). Additionally, the primary furanone detected was dibenzofuran, the primary aldehydes were lilac aldehyde A and 2-hexenal, and the main sulfur compounds and terpenes were dimethyl disulfide, beta-myrcene, gamma-terpinene, dl-panlactone, and d-limonene. Ketones and long-chain fatty acids such as 2-heptanone, 2-pentanone, and *n*-decanoic acid were also detected. Because these volatiles have low sensory thresholds, other low-abundance volatiles may also contribute significantly to their distinctive aroma [11]. These compounds are the main contributors to the strawberry-like aroma, imparting both fruity and caramel-like notes [11,27]. In summary, these compounds defined the dominant and characteristic sensory impressions of strawberry wine and juice.

Esters are a group of volatile constituents present in fruits and vegetables, and those with oral and fruity aromas are the main constituents of strawberry wine [11]. A comparison of the relative content of VOCs in strawberry wines and juices is shown in Table 4, where the total ester content differed significantly among the different samples. Among the wines, TCL had the highest relative amount of esters (37.77 µg/L), whereas ZJ had the lowest (19.27 µg/L), which matched the sensory evaluation results shown in Figure 2. Among the juices, BX_J had the highest relative amount of esters (31.36 µg/L), and TZ_J had the lowest (21.57 µg/L). The most abundant esters in strawberry wine are ethyl octanoate, ethyl decanoate, isoamyl acetate, and ethyl laurate, which are characteristic VOCs [11,15]. In general, strawberry wines contain the largest variety and the highest concentrations of ethanol esters, whereas Merlot wines contain higher concentrations of acetate esters than other types of esters [28].

Additionally, the ZJ wine had the highest relative amount of alcohol (38.81 µg/L), whereas TCL had the lowest (21.80 µg/L). Alcohols are important aromatic constituents of fermented beverages because they contribute substantially to odor intensity, although they generally tend to impart unpleasant odors [29]. Furans, ketones, aldehydes, and aromatic species were also detected in all four wines (Table S1), albeit in low amounts. These VOCs may play complementary, coordinated, and modifying roles in the aroma and sensory characteristics of strawberry wines [24].

#### 2.3.2. Principal Component Analysis and Orthogonal Partial Least Square-Discriminant Analysis of the Volatile Organic Compounds

Unconstrained principal component analysis (PCA) and constrained orthogonal partial least squares discriminant analysis (OPLS-DA) were performed to characterize the differences and similarities in VOCs among the juices and wines made from the four cultivars. PCA transforms component data into comprehensive indicators via dimensionality reduction and determines the weight of key components according to their correlations and degrees of variation [30]. As shown in Figure 2D, the combined interpretation rate of PC1 and PC2 was 36.2%, indicating that the differences in VOCs among the samples could be effectively described. The four types of strawberry juice and wine were separated without significant overlap, indicating that the samples from the different cultivars could be well distinguished. The VOCs differed significantly between the juice and wine samples, with the sample points located in different quadrants and regions. Sample points BX and BX_J, which were in the first and second quadrants, respectively, were relatively far from the other sample points, indicating that their differences from the other cultivars were greater.

As shown in Figure 2E, the OPLS-DA results were generally consistent with the PCA results (Figure 2D), with strawberry juice and wine samples located in the upper and lower quadrants, respectively. The BX wine samples were located in the first quadrant, whereas TZ, TCL, and BX were in the fourth quadrant. The distances between them were relatively close, indicating that their VOC components were relatively similar. Overall, the differences in the VOCs in strawberry juices and wines were obvious, and PCA and OPLS-DA could clearly distinguish the juices and wines of the four strawberry cultivars based on their VOCs.

### 2.4. Comparison of Key Differential Volatile Organic Compounds and Their Relative Odor Activity Values in the Strawberry Juices and Wines

#### 2.4.1. Comparison of the Quantities and Relative Amounts of Key Differential Volatile Organic Compounds in Strawberry Juices

**Figure 3:**
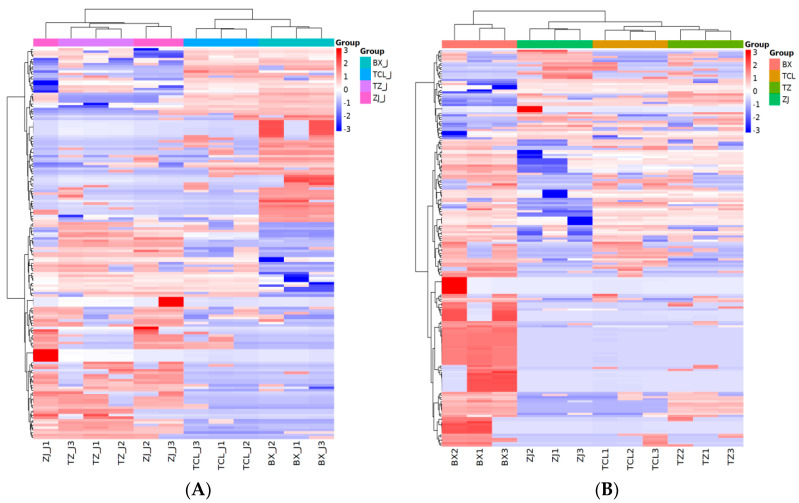
Cluster heat map of key differential VOCs in strawberry juices (**A**) and wines (**B**). Adjusted *p* < 0.05 (Tukey’s test).

Figure 3 shows the relative amounts of VOCs in strawberry juice and wine, with differences indicated by different colors, where a darker red color indicates a higher relative amount and a darker blue color indicates a lower relative amount. The inner columns represent the samples, the rows represent the metabolites, and the cluster tree on the left represents the clustering of the differential VOC species. In BX_J, the levels of 1-hexanol, 1-butanol-3-methyl-acetate, 5-methylhexanoic acid, 2-pentanone, and 2,3-butanedione were relatively high. In contrast, butanedioic acid diethyl ester, 2-pentanone, ethyl-5-methylhexanoate, 2-ethyl-1-propanol, 2-methyl-butanoic acid, and linalool levels were relatively high in BX wine. In the ZJ wine, the levels of linalool, 2-ethyl-1-propanol, ethyl-2-hydroxy-3-phenylpropanoate, butanedioic acid diethyl ester, and 2,3-butanedione were relatively high, imparting cheesy, floral, and flowery aromas to the wine [11,23].

Among strawberry wines, ZJ had a significantly higher alcohol content, TCL had higher amounts of esters and ketones, and BX had a significantly higher carboxylic acid content (Table 3, Figure 3B). Among the four strawberry juices, BX_J was rich in aldehydes and carboxylic acids (Figure 3A), making it the most suitable for fresh consumption. However, the ZJ wine had a relatively high content of different VOCs (Figure 3B), indicating that the ZJ cultivar may be more suitable for strawberry wine fermentation.

Additionally, among the differential VOCs, 157 were common in strawberry juice and 190 were common in strawberry wine, and the number of differential VOCs increased after fermentation (Tables S1 and S2). The differential VOCs may be an important reason for the differences in sensory flavors between strawberry wines and juices. However, microbial strains, inoculation methods, and fermentation processes may also influence VOCs during the fruit winemaking process [11,14,23,31]. Therefore, studies on the optimal fermentation processes are needed after suitable cultivars for strawberry wine production have been identified [2,5,23,32].

#### 2.4.2. Comparison of Relative Odor Activity Values of Key Differential Volatile Organic Compounds in Strawberry Wines

**Figure 4:**
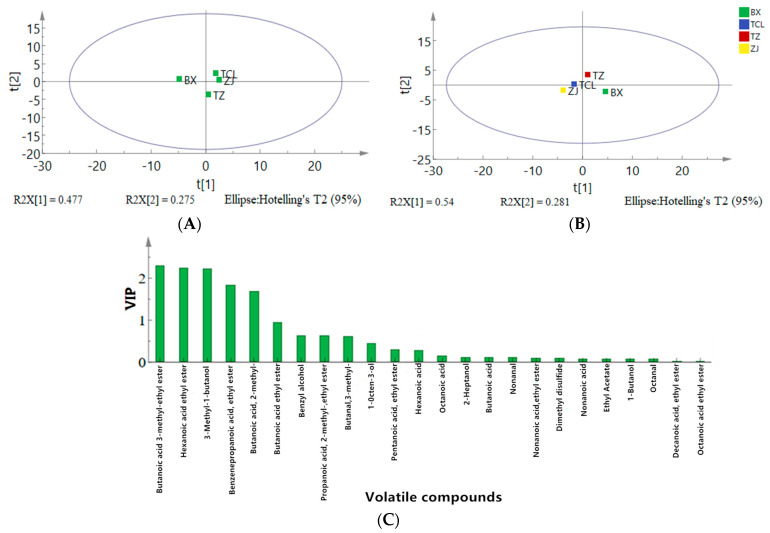
Coordinate and variable importance in projection (VIP) analyses based on the relative odor activity values of key differential VOCs in strawberry wines of different cultivars. (**A**) PCA. (**B**) OPLS-DA. (**C**) VIP value.

In the PCA, the combined interpretation rates of PC1 and PC2 reached 47.7% and 25.5%, respectively (Figure 4A), indicating differences in VOCs among the samples. The OPLS-DA results were consistent with the PCA results (Figure 4B) with strawberry wine samples located in different quadrants.

In the selection of key differential VOCs, those with a variable importance in projection (VIP) value exceeding one are usually considered the key volatiles of the group. The VIP value represents the weight ascribed to the variables within the PLS-DA model, where a higher value correlates with a more substantial contribution magnitude. Generally, VIP values exceeding 1 indicate pivotal differentiating components among samples, whereas values below 1 indicate negligible influence on differentiation [33]. As shown in Figure 4C, the VIP values of six volatile aroma components (butanoic acid ethyl ester, hexanoic acid, 3-methyl-1-butanol, benzenepropanoic acid ethyl ester, butanoic acid 3-methyl-ethyl ester, and butanedioic acid diethyl ester) exceeded 1 (*p* < 0.05). The VIP values were calculated using OPLS-DA based on the relative odor activity values (ROAVs). The differences in VOCs among strawberry juices and wines were statistically significant and were the key indicators of the differences in volatile aroma components in the wines of the different cultivars. Moreover, together with the aroma characteristics of these six aroma compounds, we found that fruit aroma was the main flavor component in strawberry wine (Table S3), indicating that these six compounds could influence the differences between strawberry wine samples, which is similar to the results of the sensory evaluation.

Five key differential VOCs with ROAVs exceeding 1 (butanoic acid 3-methyl-ethyl ester, 3-methyl-1-butanol, hexanoic acid ethyl ester, butanoic acid ethyl ester, and octanoic acid ethyl ester) played important roles in the sensory features of the strawberry juice and wine samples (Table S3). Moreover, six key differential VOCs with ROAVs exceeding 1 (the five key differential VOCs listed above and 2-methyl-propanoic acid ethyl ester) played important roles in the sensory features of the strawberry wine samples (Table S3).

#### 2.4.3. Network Diagram of the Relationships between Various Volatile Organic Compounds for Imparting Unique Sensory Flavor Characteristics in Strawberry Wines

**Figure 5:**
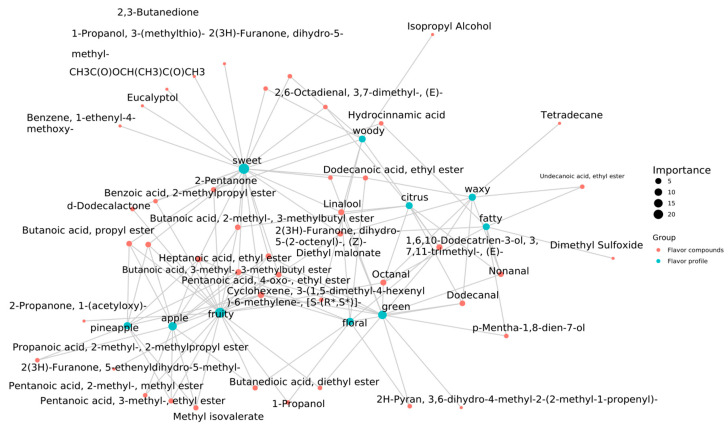
Network showing the relationships between the sensory flavor characteristics and flavor substances of strawberry wines.

With the FlavorDB2 database [34], the iGraph tool was used to build a network diagram of the relationships between flavor substances to impart unique sensory flavor characteristics. In Figure 5, the green circles represent sensory features, and the red circles represent flavor compounds. A larger green circle indicates that more types of flavor compounds are associated with that sensory feature, rendering that feature more important. By contrast, a larger red circle indicates that more sensory features are associated with the flavor compound, rendering it an important flavor substance. By default, the top 10 sensory features were used to create a network diagram.

As shown in Figure 5, fruity, sweet, apple, green, and floral sensory features were associated with more than nine types of flavor compounds. For example, the fruity sensory feature was associated with butanoic acid propyl ester, 3-methyl butyl ester, 1-propanol, and 19 other flavor compounds. Floral sensory features were associated with dodecanoic acid ethyl ester, linalool, 1-propanol, and six other flavor substances. Overall, substances related to fruit aroma were the main flavor components of strawberry wine. At the same time, we also noticed that the flavor compound linalool was associated with floral, sweet, green, woody, and citrus sensory features. Linalools exhibit antimicrobial, anti-inflammatory, anticancer, and antioxidative properties [35]. However, linalool was not detected in strawberry juice, indicating that it might be produced by yeast metabolism during fermentation [36].

Few studies have examined the correlation between cultivar origin, sensory quality, fermentation process, and characteristics of different strawberry cultivars [4]. Sensory evaluations based on consumer preferences are indispensable for the production and marketability of strawberry wine. Given that VOCs significantly influence sensory attributes, a comprehensive analysis using GC-MS combined with electronic tongue, electronic nose, and sensory evaluations is vital for ensuring the quality of strawberry wines.

## 3. Materials and Methods

### 3.1. Samples and Reagents

#### 3.1.1. Strawberry Cultivars and Yeast Strain for Brewing

Four strawberry cultivars, named Snow White (BX), Sweet Charlie (TCL), Tongzhougongzhu (ZJ), and Zhangji (Akihime, ZJ), were collected in Wenji Town (N: 33°03′22.50″, E: 115°38′43.60″; Fuyang City, Anhui Province, China) between March and April 2022. These four typical strawberry cultivars are widely cultivated in China, and the sampled strawberry fruits had a normalized shape, 90% maturity, no damage, and approximately the same size, and were stored at −20 °C after screening until processing for juice press and fermentation. The yeast strain Lalvin Rhône 2323 (Lallemand Inc., Montreal, Canada) was used to brew the strawberry wine.

#### 3.1.2. Strawberry Juice Preparation and Wine Fermentation

Frozen strawberry fruit (5 kg) was thawed at 25 °C for 2 h and then crushed using a juice press. Samples (5 mL) of the juice were collected for GC × GC-TOF-MS analysis of VOCs. The juices were designated as ZJ_J, TCL_J, BX_J, and TZ_J, and each sample was tested three times.

Before the fermentation process, 30 ppm of SO_2_ and 300 mg of the commercial pectinase Lallzyme EX-V (30 mg/L) were added to the raw juice, and the mixture was incubated for 2 h at 40 °C [15]. The enzyme-treated juice was centrifuged at 8000× *g* for 10 min at 4 °C and the Brix was then adjusted to 23° with sucrose [31]. The mixture was pasteurized for 30 s in a hot water bath at 97 °C and then immediately cooled to ambient temperature in an ice bath. For fermentation, 300 mL of pasteurized strawberry juice was immediately transferred to a 500 mL sterile jar [4].

The Lalvin Rhône 2323 yeast strain was activated for 12 h at 28 °C in yeast extract-peptone-dextrose liquid medium and diluted strawberry juice (with purified water), and 1.0% (*v*/*v*) strawberry juice preculture was inoculated into the pasteurized juice to start fermentation. The fermentation temperature was controlled at 25 °C (± 2 °C) [37]. During brewing, Brix was measured every 2 days until there was no change in three consecutive measurements, which signaled the completion of alcoholic fermentation [4]. Then, 5 mL samples of fermented strawberry wine were used for VOC analysis. The wines were designated ZJ, TCL, BX, and TZ, and each sample was tested three times.

#### 3.1.3. Chemicals and Reagents

Typical C7 to C30 alkanes (≥99.8%) were purchased from Sigma (St. Louis, MO, USA). Analytical-grade sodium chloride was purchased from Sinopharm (Shanghai, China), and chromatography-grade *n*-hexyl-d13 alcohol (≥98.5%) was purchased from C/D/N Isotopes (Quebec, QC, Canada). Chromatography-grade anhydrous ethanol (≥99.8%) was purchased from Aladdin (Shanghai, China). Ultrapure water was obtained using a Milli-Q system (Millipore, Billerica, MA, USA).

### 3.2. Descriptive Sensory Analysis

Sensory analysis was performed using the methods described by Zhao et al. [20], and wine samples were assessed by a panel of eight experts (four females and four males, between 25 and 40 years of age, from Anhui Jinzhongzi Distillery, Anhui, China), all of whom had more than 5 years of experience in the sensory evaluation of various drinks (including Baijiu and wines). Before carrying out the sensory evaluation of the strawberry wines, the eight experts underwent training for 4 h per week over 2 months using the “Le Nez du Vin” aroma kit (54 aromas; Yixiangle, Hong Kong, China) [15]. Sensory evaluation was performed in a well-ventilated, odor-free, quiet room, and a mouthwash was provided to the panelists during the evaluation.

The sensory qualities of the four strawberry wine varieties were analyzed quantitatively and descriptively. The evaluators discussed and identified the 18 descriptors and their corresponding reference samples. Descriptive terms for color, six aroma attributes, eight taste attributes, and three overall evaluations of the wine body were scored on a scale of 0–9 points (0–2, very weak; 3–5, medium; and 6–9, very strong) [38]. Each panelist individually scored the samples in the order mentioned above, and a mean score was obtained [20,38].

### 3.3. Physicochemical Parameter Detection

The °Brix and pH values were measured using a refractometer (ATGO, Tokyo, Japan) and pH meter (FE28, Mettler Toledo, Greifensee, Switzerland), respectively. A fully automatic analyzer (Y15, BioSystem, Barcelona, Spain) and relevant chemical kits were used to analyze the total acid content. The glucose and ethanol contents were determined using high-performance liquid chromatography on an LC-16 system (Shimadzu, Kyoto, Japan) equipped with an RID-20A refractive index detector and a Bio-Rad Aminex HPX-87H resin-based column (300 × 7.8 mm) at 55 °C, with 5 mM H_2_SO_4_ used for elution at a flow rate of 0.5 mL/min [39].

### 3.4. GC × GC–TOF-MS Analysis of Volatile Organic Compounds

#### 3.4.1. Preparation of Internal Standard Solution

An appropriate amount of *n*-hexyl-d 13 alcohol was transferred to a volumetric flask, dissolved in 50% (*v*/*v*) ethanol solution to a final concentration of 10 mg/L, and stored at 4 °C. A stock solution with 10 mg/L of *n*-alkanes was prepared in *n*-hexane and stored at 4 °C.

#### 3.4.2. HS-SPME Method

An appropriate amount of sample was transferred to a 15 mL glass test tube and diluted to a 10% ethanol concentration (*v*/*v*) with an aqueous solution of saturated NaCl [40]. Then, 5 mL of each diluted sample was transferred to a 20 mL headspace vial. Subsequently, 10 µL of the internal standard solution was added to each sample, and the mixtures were incubated at 50 °C for 10 min. The samples were extracted using a headspace solid-phase microextracter coated with a DVB/CAR/PDMS fiber head (50/30 µm × 1 cm; Supelco, Bellefonte, PA, USA) and incubated at 50 °C for 20 min.

The extracted samples were desorbed in the GC injection port at 250 °C for 5 min before GC × GC–TOF-MS analysis using set parameters [41,42,43]. After the injection step, SPME fiber was placed in the chamber at 270 °C for 10 min. Then, 10 μL of the *n*-alkanes was transferred to a 20 mL headspace vial and extracted and injected into the GC port as described above. Each sample was subjected to three parallel tests to ensure the reproducibility of the experimental results.

#### 3.4.3. GC × GC–TOF-MS Method

GC × GC-TOF-MS analyses were performed using a LECO Pegasus 4D instrument (LECO, St. Joseph, MI, USA) consisting of an Agilent 8890A GC system (Agilent Technologies, Palo Alto, CA, USA) equipped with a split/splitless injector and dual-stage cryogenic modulator (LECO) coupled to a TOF-MS detector (LECO). A DB-Heavy Wax column (30 m × 250 μm I.D., 0.5 μm; Agilent) was used for the first dimension, and a Rxi-5Sil MS column (2.0 m × 150 μm I.D., 0.15 μm; Restek, Bellefonte, PA, USA) for the second dimension.

For GC × GC, high-purity helium (>99.999%) was used as a carrier gas at a constant flow rate of 1.0 mL/min. The temperature program of the oven was as follows: the oven temperature was first held at 40 °C for 3 min, then raised to 200 °C at the rate of 6 °C/min, then raised to 250 °C at the rate of 10 °C/min, and finally held for 5 min. The second oven temperature was set to 5 °C higher than that of the first oven. The temperature of the modulator was always 15 °C higher than that of the second column. The modulator was operated with a 4.0 s modulation period. The GC injector temperature was 250 °C.

For the TOF-MS, the flavor substances were analyzed on a LECO Pegasus BT 4D system, with the transfer line and TOF-MS ion source temperatures both set at 250 °C, respectively. The acquisition frequency was 200 spectra/s. The mass spectrometer was operated in electron impact ionization mode at 70 eV using an *m*/*z* range of 35–550 and a detector voltage of 1960 V [41,42,43].

### 3.5. Statistical Analysis

All measurements were performed in triplicate and presented as mean values and standard deviations. The statistical significance of the data was evaluated via variance analysis by comparative averages (ANOVA) using the Duncan test and SPSS 22.0, program for Windows (IBM, New York, NY, USA).

#### 3.5.1. Data Processing

Data were collected using a Pegasus 4D workstation (LECO) and analyzed and processed using the Chroma TOF software program built into the instrument. This program automatically identified peaks with a signal-to-noise ratio of over 50 and compared them with the NIST 14 and Wiley 9 MS libraries to automatically generate a peak table. Compounds with halogen and silicon elements were removed, and chromatographic peaks with more than 700 forward and reverse similarities were screened using MS [18].

The retention index (RI) of each VOC was calculated for C7–C30 *n*-alkanes and compared with the reference RI values in the NIST online database (https://webbook.nist.gov/; accessed on 21 September 2022). RI differences of 50 or less were selected, and compounds with greater than 50% occurrence rates were considered reliable results [44]. The internal standard method was used to calculate the content of each flavor compound, as shown in Equation (1).

(1) $$Volatile\;organic\;compound\;content\;(µg/L)=\frac{Peak\;area\;of\;compound\times Internal\;standard\;amount\;(µg/L)}{Peak\;area\;of\;internal\;standard}$$

The ROAVs were used to estimate the contribution of aromatic compounds to the overall flavor of the wine. VOCs with ROAVs exceeding 1 were generally the key flavor compounds, whereas those with ROAVs between 0.1 and 1 were not considered important for the overall flavor of the sample [45]. ROAVs were calculated by dividing the relative concentration of an aromatic compound by its odor threshold value. The largest defined group was divided into a ROAV of 100, and the ROAVs of the other substances were corrected according to Equation (2):

ROAV = (Peak B/TB)/(Peak A/TA)(2)

where Peak A is the maximum peak area of the component, Peak B is the peak area of the material to be measured, TA is the odor value of the largest component, and TB is the odor value of the compound to be measured.

#### 3.5.2. Chemometric Analysis of the GC × GC–TOF-MS Data

GC × GC-TOF-MS data were mined using a chemometric method, and irrelevant and redundant variables were filtered through preprocessing. The variables were analyzed semi-quantitatively based on internal standards. A small portion of the missing values was interpolated using the K-nearest neighbor algorithm based on machine learning to simplify the univariate and multivariate analyses. Variables with no statistical significance were removed by univariate analysis, and potentially important compounds were preliminarily screened. Single-factor analysis of variance (ANOVA) was performed using SPSS software (version 22.0; IBM, New York, NY, USA).

The error detection rate was used to correct the *p*-values to reduce false-positive results, and the variables were preliminarily screened on the basis of a *p*-value of less than 0.05 and a Pearson correlation coefficient |r| greater than 0.6. Data selected by one-way ANOVA were used for multivariate statistical analyses after scaling by unit variance. PCA and OPLS-DA were performed using SIMCA-P 14.1 software. Cluster heat and radar maps were plotted using the BioDeep online analysis platform (https://www.biodeep.cn/, accessed on 16 August 2024.). The correlative network of data pairs satisfying |*ρ*| > 0.6 and *p* < 0.05 was visualized with Gephi (Version 0.9.1).

## 4. Conclusions

This study was conducted to reveal the VOC characteristics of the juices and wines of four typical strawberry cultivars and to distinguish the sources and varieties of strawberry raw materials for wine fermentation. The findings revealed that the samples had obvious differences in VOC content, and PCA and OPLS-DA of the VOCs could distinguish strawberry juices and wines of the four different cultivars. In strawberry wines, six VOCs had VIP values exceeding 1: butanoic acid ethyl ester, hexanoic acid, 3-methyl-1-butanol, benzenepropanoic acid ethyl ester, butanoic acid 3-methyl-ethyl ester, and butanedioic acid diethyl ester. Overall, the ZJ wine had a relatively high alcohol content (13.25 ± 0.59%, *v*/*v*) and sensory evaluation score, indicating that the ZJ cultivar is more suitable for fermentation. The main factors affecting the differences in flavor-related VOCs in wine include cultivars and cultivation techniques, fermentation strains and processes, storage containers and conditions, and aging times and processes. Further studies will be conducted to determine the effects of these factors on the quality of strawberry wine with the aim of providing reference data for the future production of such fruit wines.

## Figures and Tables

**Table 1:** Ethanol and residual sugar contents in fermented juices of four different strawberry cultivars.

Cultivar/Sample	Ethanol (%, *v*/*v*)	Residual Sugar (g/L)
BX	13.25 ± 0.59	3.47 ± 0.65 ^a^
TCL	13.02 ± 0.6	3.44 ± 0.17 ^a^
TZ	13.12 ± 0.08	1.86 ± 0.02 ^b^
ZJ	13.69 ± 0.1	3.22 ± 0.04 ^a^

Note: Means ± standard deviations (*n* = 3) followed by different letters within each row indicate significant differences (Duncan’s test, *p* < 0.05). BX, Snow White; TCL, Sweet Charlie; TZ, Tongzhougongzhu; ZJ, Akihime.

**Table 2:** pH values and total acid contents of wines and juices produced from four different strawberry cultivars.

Cultivar/ Sample	pH		Total Acid (g/L)	
	Juice	Wine	Juice	Wine
BX	4.06 ± 0.05 ^a^	4.03 ± 0.05 ^a^	4.03 ± 0.05 ^d^	5.46 ± 0.37 ^b^
TCL	3.36 ± 0.05 ^d^	3.53 ± 0.05 ^d^	6.63 ± 0.15 ^a^	7.7 ± 0.1 ^a^
TZ	3.7 ± 0 ^b^	3.83 ± 0.05 ^b^	6.26 ± 0.05 ^b^	7.53 ± 0.23 ^a^
ZJ	3.6 ± 0 ^c^	3.73 ± 0.05 ^c^	5.46 ± 0.32 ^c^	6.56 ± 0.11 ^b^

Note: Means ± standard deviations (*n* = 3) followed by different letters within each row indicate significant differences (Duncan’s test, *p* < 0.05). BX, Snow White; TCL, Sweet Charlie; TZ, Tongzhougongzhu; ZJ, Akihime.

**Table 3:** Quantification of VOCs detected in strawberry wines and juices using GC × GC–TOF-MS.

Group		Ketones	Hydrocarbons	Heterocyclic Compounds	Aldehydes	Esters	Alcohols	Carboxylic Acids	Others	Total
Wine	BX	48	88	16	9	130	82	35	478	886
	TCL	47	74	15	6	125	86	30	441	824
	TZ	43	73	20	9	121	79	30	434	809
	ZJ	40	58	23	9	100	73	27	382	712
Juice	BX_J	63	109	26	20	123	96	39	552	1028
	TCL_J	61	90	22	10	103	90	16	456	848
	TZ_J	53	88	16	10	132	84	20	456	859
	ZJ_J	63	90	11	17	112	102	24	499	918

**Table 4:** Comparison of the relative amounts of VOCs between strawberry wines and juices (µg/L).

Group		Alcohols	Aldehydes	Carboxylic Acids	Esters	Heterocyclic Compounds	Hydrocarbons	Ketones	Others
Wine	BX	29.77	0.09	5.82	27.92	5.78	1.14	0.81	28.62
	TCL	21.80	0.14	5.51	37.77	0.25	1.58	1.40	31.50
	TZ	29.75	0.10	3.78	28.38	1.56	2.30	1.01	33.09
	ZJ	38.81	0.16	5.09	19.27	6.30	0.51	0.84	29.00
Juice	BX_J	18.82	0.69	7.16	31.36	0.53	1.18	3.12	37.11
	TCL_J	22.19	0.20	8.98	18.13	3.37	1.47	2.23	43.40
	TZ_J	26.37	0.08	1.79	21.57	0.28	0.63	4.26	44.98
	ZJ_J	17.44	0.64	3.72	21.99	0.07	2.58	6.45	47.07

## Data Availability

The original contributions presented in the study are included in the article/Supplementary Materials, further inquiries can be directed to the corresponding author.

## Supplementary Materials

The following supporting information can be downloaded at: https://www.mdpi.com/article/10.3390/molecules29194691/s1, Table S1. Classification and relative content of different VOCs in strawberry wine samples. Table S2. Classification and relative content of different VOCs in strawberry juice samples. Table S3. ROAV values of different VOCs in samples.
