# Supplementary material for: Comparison of Flavor Differences between the Juices and Wines of Four Strawberry Cultivars Using Two-Dimensional Gas Chromatography-Time-of-Flight Mass Spectrometry and Sensory Evaluation
Source: Molecules. 2024 Oct 3;29(19):4691. doi: 10.3390/molecules29194691 (PMC11477698; doi:10.3390/molecules29194691)
Supplement: Supplementary file 1 [file molecules-29-04691-s001.zip › Table S2. Classification and relative content of different VOCs in strawberry juice samples..docx]

**Table S2.** Classification and relative content of different VOCs in strawberry juice samples.

| **Group** | | Contents /（μg/L） | | | |
| --- | --- | --- | --- | --- | --- |
|  |  | BX-J | TCL-J | TZ-J | ZJ-J |
| **Ketones** | 1-Octen-3-one | n.d. | n.d. | 0.1±0 | 0.2±0.03 |
|  | 1-Pentanone, 1-(4-methylphenyl)- | n.d. | n.d. | n.d. | 0.04±0 |
|  | 2-Buten-1-one, 1-(2,6,6-trimethyl-1,3-cyclohexadien-1-yl)- | n.d. | n.d. | 0.06±0.02 | n.d. |
|  | 2-Butyl-3-(4-beta-diethylaminoethoxybenzoyl)benzofuran | 0.08±0.01 | 0.12±0.04 | n.d. | n.d. |
|  | 2-Hexanone | n.d. | n.d. | 0.63±0.06 | 0.24±0.2 |
|  | 2-Hydroxy-iso-butyrophenone | n.d. | 0.02±0 | 0.05±0 | 0.03±0 |
|  | 2-Octen-4-one | 0.50±0.06 | n.d. | n.d. | n.d. |
|  | 2-Pentanone | 11.28±0.26 | 8.97±0.17 | 56.36±2.2 | 43.14±0.39 |
|  | 2-Propanone, hydrazone | n.d. | 0.11±0 | 0.30±0.02 | 0.45±0.04 |
|  | 2-Undecanone | n.d. | n.d. | 0.53±0.02 | 0.72±0.02 |
|  | 3-Octanone | 0.45±0.06 | 1.27±0.19 | 0.66±0.12 | n.d. |
|  | 3-Pentanone, 2-methyl- | n.d. | 0.13±0.02 | 0.05±0 | 0.10±0.01 |
|  | 3-Penten-2-one | 3.51±0.25 | 1.50±0.1 | 0.61±0.05 | 0.11±0.01 |
|  | 3-Penten-2-one, 3,4-dimethyl- | n.d. | n.d. | 0.44±0.03 | 0.73±0.01 |
|  | 3,3-Dimethylcyclohexanone | 0.02±0 | 0.02±0 | n.d. | n.d. |
|  | 4'-(Trifluoromethyl)acetophenone | n.d. | n.d. | 0.04±0 | 0.12±0 |
|  | CH3C(O)CH2CH2OH | 0.09±0.01 | 0.09±0.01 | n.d. | n.d. |
|  | Cyclopentanone, 2,2,4-trimethyl- | n.d. | n.d. | n.d. | 0.03±0 |
|  | Ethanone, 1-(4-methylphenyl)- | 0.05±0 | 0.10±0.01 | 0.06±0.01 | n.d. |
| **Hydrocarbons** | 2,4,6-Octatriene, 2,6-dimethyl-, (E,Z)- | n.d. | n.d. | 0.26±0.03 | 0.31±0.13 |
|  | 5-Ethyldecane | 0.07±0.04 | 0.09±0.01 | n.d. | n.d. |
|  | Bicyclo[2.2.1]hept-2-ene, 1,7,7-trimethyl- | 0.13±0.03 | 0.16±0.02 | 0.25±0.03 | 0.18±0.03 |
|  | Nonane, 3-methyl- | 0.08±0.02 | 0.11±0.02 | n.d. | 0.02±0 |
|  | Tridecane, 5-methyl- | n.d. | 0.07±0.01 | 0.21±0.01 | 0.07±0.01 |
|  | Undecane | 0.05±0.02 | 0.07±0.01 | n.d. | n.d. |
|  | Undecane, 2-methyl- | n.d. | 0.11±0.05 | n.d. | 0.17±0 |
|  | Undecane, 3-methylene- | 0.08±0.01 | 0.09±0.01 | 0.07±0.02 | n.d. |
| **Heterocyclic_Compounds** | 2(3H)-Furanone, 5-ethenyldihydro-5-methyl- | 0.30±0.03 | 0.05±0.01 | 0.03±0 | n.d. |
|  | 3-Furancarboxylic acid, methyl ester | 0.02±0 | n.d. | n.d. | n.d. |
|  | 3(2H)-Isoxazolone, 4,5-dimethyl- | 8.16±2.43 | 1.12±0.14 | n.d. | 1.32±0.8 |
|  | 4-Vinyl-imidazole | 0.38±0.02 | n.d. | n.d. | n.d. |
|  | 5-Oxotetrahydrofuran-2-carboxylic acid | 0.39±0.01 | 0.57±0.08 | 0.17±0.02 | 0.09±0.03 |
|  | Ethyl 3-methyl-1H-pyrazole-4-carboxylate | 0.01±0 | n.d. | n.d. | n.d. |
|  | Thiophene, 2,5-bis(1,1-dimethylethyl)- | 0.05±0 | n.d. | n.d. | n.d. |
| **Aldehydes** | 1H-Indole-3-carboxaldehyde | n.d. | 0.03±0 | 0.05±0.01 | 0.03±0 |
|  | 3-(4-Isopropylphenyl)-2-methylpropionaldehyde | n.d. | n.d. | n.d. | 0.03±0 |
|  | Benzoic acid, 2-formyl- | n.d. | n.d. | 1.16±0.27 | 0.57±0.29 |
|  | Butanal, 2-methyl- | 0.39±0.04 | 1.38±0.03 | n.d. | n.d. |
|  | Butanal, 3-methyl- | 0.44±0.02 | 0.68±0.06 | 1.63±0.11 | 3.59±0.04 |
| **Esters** | 1-Butanol, 3-methyl-, acetate | 16.17±1.35 | 17.16±0.87 | 55.94±7.12 | 3.21±1.28 |
|  | 2-Butenoic acid, methyl ester | n.d. | n.d. | 7.79±0.24 | 5.47±0.27 |
|  | 2-Butenoic acid, methyl ester, (E)- | 2.22±0.02 | 0.88±0.08 | n.d. | n.d. |
|  | 2-Ethyl-n-butyric acid ethyl ester | 0.22±0.01 | n.d. | n.d. | n.d. |
|  | 2-Pentenoic acid, ethyl ester | 0.10±0.01 | n.d. | n.d. | n.d. |
|  | 2,2,4-Trimethyl-1,3-pentanediol diisobutyrate | n.d. | 2.28±0.52 | 4.67±0.2 | 7.41±1.32 |
|  | 2(3H)-Furanone, dihydro-5-propyl- | 0.16±0.02 | n.d. | n.d. | n.d. |
|  | 3-Hexen-1-ol, acetate, (Z)- | n.d. | n.d. | 0.67±0.12 | 1.08±0.64 |
|  | Acetic acid, heptyl ester | n.d. | 0.05±0 | 5.58±1.23 | 0.07±0.02 |
|  | Acetic acid, nonyl ester | n.d. | n.d. | 1.33±0.35 | 0.16±0.03 |
|  | Acetic acid, pentyl ester | 0.71±0.07 | 0.95±0.1 | 0.96±0.16 | 0.31±0.04 |
|  | Benzenepropanoic acid, ethyl ester | 1.29±0.01 | 0.09±0.01 | 1.73±0.44 | 0.03±0 |
|  | Butanoic acid, 1-methyloctyl ester | n.d. | 0.01±0 | n.d. | 0.02±0 |
|  | Butanoic acid, 2-methyl-, 3-methylbutyl ester | 0.54±0.08 | 0.05±0.01 | n.d. | n.d. |
|  | Butanoic acid, 3-methyl-, 3-methylbutyl ester | 0.83±0.15 | n.d. | n.d. | 0.02±0 |
|  | Dodecanoic acid, methyl ester | n.d. | 0.37±0.01 | 0.64±0.02 | 0.09±0.01 |
|  | Ethyl 5-methylhexanoate | 2.76±0.26 | n.d. | n.d. | n.d. |
|  | Isopropyl butyrate | n.d. | n.d. | 0.97±0.12 | 1.96±0.05 |
|  | Methyl isobutyrate | n.d. | n.d. | 0.13±0.02 | 0.07±0 |
|  | n-Propyl benzoate | 0.16±0.01 | n.d. | n.d. | n.d. |
|  | n-Propyl cinnamate | 0.09±0 | n.d. | n.d. | n.d. |
|  | Pentanoic acid, 2-methyl-, methyl ester | n.d. | n.d. | n.d. | 0.13±0.01 |
|  | Propanoic acid, 2-methyl-, 1-methylbutyl ester | 0.22±0.01 | n.d. | n.d. | n.d. |
|  | Propanoic acid, 2-methyl-, 2-(hydroxymethyl)-1-propylbutyl ester | 0.14±0.01 | n.d. | n.d. | n.d. |
|  | Propanoic acid, 2-methyl-, 3-methylbutyl ester | 0.10±0.01 | n.d. | n.d. | n.d. |
| **Alcohols** | (Z)-4-Decen-1-ol | n.d. | 0.25±0.02 | 0.16±0.02 | 0.58±0.02 |
|  | 1-Butanol | 1.61±0.02 | 1.09±0.14 | 1.35±0.06 | 0.27±0 |
|  | 1-Decanol | 0.04±0 | 0.32±0.32 | 9.95±2.03 | 2.60±0.28 |
|  | 1-Heptanol | 1.05±0.12 | 4.62±0.76 | 5.57±0.25 | 4.33±0.01 |
|  | 1-Heptanol, 2-propyl- | 0.21±0.01 | 0.17±0 | 0.45±0.07 | 0.39±0.02 |
|  | 1-Hexanol | 39.54±0.68 | 39.23±3.88 | 106.87±8.91 | 105.86±1.61 |
|  | 1-Hexanol, 4-methyl- | n.d. | n.d. | 0.12±0.01 | 0.20±0 |
|  | 1-Octanol | 2.53±0.06 | 12.39±1.64 | 114.67±2.51 | 37.49±2.17 |
|  | 1-Pentanol | 1.74±0.03 | 2.73±0.3 | 1.61±0.05 | 1.16±0.22 |
|  | 2-Decanol | n.d. | n.d. | 0.16±0.02 | 0.05±0.01 |
|  | 2-Nonanol | 0.08±0.01 | 1.65±0.16 | 25.95±4.36 | 3.09±0.15 |
|  | 2-Penten-1-ol, (E)- | 0.07±0 | n.d. | n.d. | n.d. |
|  | 2,4,7,9-Tetramethyl-5-decyn-4,7-diol | n.d. | 0.12±0.01 | 0.33±0.08 | 0.15±0.02 |
|  | 3-Hexen-1-ol, 2-ethyl- | 0.05±0 | n.d. | n.d. | n.d. |
|  | 3-Nonen-1-ol, (Z)- | 0.13±0.02 | 0.27±0.03 | 0.27±0.04 | 0.33±0.02 |
|  | 4-Heptanol | 0.04±0 | n.d. | n.d. | n.d. |
|  | 4-Nonanol | 0.05±0.01 | 0.05±0.01 | 0.16±0.03 | n.d. |
|  | 4-Nonanol, 2,6,8-trimethyl- | n.d. | n.d. | n.d. | 0.06±0.01 |
|  | 5,9-Undecadien-2-ol, 6,10-dimethyl- | n.d. | n.d. | n.d. | 1.09±0.11 |
|  | 7-Octene-2,6-diol, 2,6-dimethyl- | n.d. | n.d. | 0.03±0 | 0.02±0 |
|  | cis-Hept-4-enol | 0.03±0 | 0.31±0.04 | 0.30±0.03 | 0.13±0 |
|  | dl-Menthol | 0.07±0001 | n.d. | n.d. | 0.07±0 |
|  | Eucalyptol | n.d. | 0.42±0.09 | 0.27±0.05 | 0.25±0.02 |
|  | Isopropyl Alcohol | 1.01±0.06 | 0.78±0.01 | 2.17±0.25 | 1.18±0.02 |
|  | p-Mentha-1,8-dien-7-ol | 0.13±0 | n.d. | n.d. | n.d. |
| **Carboxylic_Acids** | 2-Octenoic acid | 0.19±0.01 | 0.1±0.01 | n.d. | 0.11±0.06 |
|  | 3-Octenoic acid, (E)- | 0.34±0.01 | 0.06±0 | n.d. | 0.05±0 |
|  | 5-Methylhexanoic acid | 14.86±0.21 | 0.24±0.01 | n.d. | 0.12±0.04 |
|  | 9-Decenoic acid | n.d. | n.d. | 29.21±3.46 | 0.14±0.01 |
|  | Hexanoic acid, 3,5,5-trimethyl- | n.d. | 0.02±0 | n.d. | 0.04±0 |
|  | Hexanoic acid, anhydride | n.d. | n.d. | 0.23±0.05 | 0.25±0.05 |
|  | Hydroxyproline | 0.43±0.06 | n.d. | n.d. | n.d. |
|  | L-Alanine, 3-sulfo- | n.d. | n.d. | 0.37±0.02 | n.d. |
|  | Propanedioic acid, propyl- | 0.25±0.07 | n.d. | n.d. | n.d. |
| **Others** | 1-Heptene, 5-methyl- | n.d. | n.d. | 0.10±0.04 | 0.03±0 |
|  | 1-Octene, 6-methyl- | n.d. | n.d. | n.d. | 0.08±0 |
|  | 1-Oxaspiro[4.5]dec-6-ene, 2,6,10,10-tetramethyl- | n.d. | n.d. | 0.02±0 | 0.01±0 |
|  | 1,3-Propanediol, 2-ethyl-2-(hydroxymethyl)- | n.d. | 0.02±0.01 | 0.06±0.01 | 0.03±0 |
|  | 1,7-Octadiene, 2,7-dimethyl-3,6-bis(methylene)- | n.d. | n.d. | 0.12±0.01 | 0.07±0 |
|  | 1,8-Cyclopentadecadiyne | 0.18±0.02 | n.d. | n.d. | n.d. |
|  | 4-Vinylphenol | n.d. | n.d. | 28.39±2.59 | n.d. |
|  | a-Methylstyrene | 0.33±0 | 0.14±0.01 | 0.08±0.01 | 0.09±0.02 |
|  | Benzene, 1,1'-(1,1,2,2-tetramethyl-1,2-ethanediyl)bis- | 0.11±0.01 | n.d. | n.d. | n.d. |
|  | Benzene, 1,1'-(1,2-cyclobutanediyl)bis-, cis- | n.d. | n.d. | n.d. | 185.21±9.95 |
|  | Benzene, 1,2,4,5-tetramethyl- | n.d. | 0.05±0.01 | 0.07±0.01 | 0.05±0.01 |
|  | Benzene, 2-(2-butenyl)-1,3,5-trimethyl- | 0.04±0.01 | 0.01±0 | n.d. | 0.02±0 |
|  | Ether, hexyl isopropyl | n.d. | n.d. | 0.32±0.04 | n.d. |
|  | Ethylbenzene | n.d. | n.d. | 0.06±0.01 | 0.98±0.04 |
|  | Formic acid hydrazide | 6.62±0.84 | 0.05±0.01 | n.d. | n.d. |
|  | Methyl thiolacetate | n.d. | n.d. | 0.37±0.04 | 0.15±0.03 |
|  | Naphthalene, 1-methyl- | 0.07±0 | 0.07±0.01 | n.d. | n.d. |
|  | Naphthalene, 1,2-dihydro-1,1,6-trimethyl- | 0.02±0 | n.d. | n.d. | n.d. |
|  | Styrene | n.d. | 52.60±6.08 | 300.28±5.91 | 0.02±0 |

**Note:** n.d. Indicates that the volatile component is not detected.
