# Supplementary material for: Comparison of Flavor Differences between the Juices and Wines of Four Strawberry Cultivars Using Two-Dimensional Gas Chromatography-Time-of-Flight Mass Spectrometry and Sensory Evaluation
Source: Molecules. 2024 Oct 3;29(19):4691. doi: 10.3390/molecules29194691 (PMC11477698; doi:10.3390/molecules29194691)
Supplement: Supplementary file 1 [file molecules-29-04691-s001.zip › Table S1. Classification and relative content of different VOCs in strawberry wine samples..docx]

**Table S1.** Classification and relative content of different VOCs in strawberry wine samples.

| **Group** | | Contents /(μg/L) | | | |
| --- | --- | --- | --- | --- | --- |
|  |  | BX | TCL | TZ | ZJ |
| **Ketones** | 1-Pentanone, 1-(4-methylphenyl)- | n.d. | n.d. | 0.1±0 | 0.1±0 |
|  | 1,3,7,7-Tetramethyl-9-oxo-2-oxabicyclo[4.4.0]dec-5-ene | 0.14±0.02 | n.d. | n.d. | n.d. |
|  | 2-Pentanone | 8.61±0.33 | 5.41±0.23 | 11.68±0.05 | n.d. |
|  | 2-Pentanone, 4-hydroxy- | n.d. | 0.45±0.02 | n.d. | 0.2±0.01 |
|  | 2-Propanone, 1-(acetyloxy)- | 0.36±0.09 | n.d. | n.d. | n.d. |
|  | 2-Propanone, 1,1-dimethoxy- | n.d. | n.d. | n.d. | 0.14±0.01 |
|  | 2,3-Butanedione | n.d. | n.d. | 6.07±0.04 | 11.46±0.05 |
|  | 2,3-Octanedione | 0.26±0.02 | 0.15±0.02 | n.d. | n.d. |
|  | 3-Hexanone, 2,4-dimethyl- | 0.34±0 | 0.16±0.05 | n.d. | n.d. |
|  | 3-Methoxy-2-methyl-cyclohex-2-enone | 2.24±0.17 | n.d. | n.d. | n.d. |
|  | 3-Nonen-2-one | n.d. | 0.21±0.01 | 0.15±0 | 0.15±0.02 |
|  | 4-(2,6,6-Trimethylcyclohexa-1,3-dienyl)but-3-en-2-one | n.d. | n.d. | n.d. | 0.11±0 |
|  | 4-Nonanone | 0.24±0.04 | n.d. | n.d. | n.d. |
|  | CH3C(O)CH2CH2OH | n.d. | n.d. | 4.31±0.44 | 2.71±0.02 |
|  | CH3C(O)OCH(CH3)C(O)CH3 | 0.32±0.04 | 0.13±0 | n.d. | n.d. |
|  | Ethanone, 1-(1,3-dimethyl-3-cyclohexen-1-yl)- | 0.35±0.26 | 0.15±0.03 | 0.25±0.01 | n.d. |
|  | Ethanone, 1-(3-methylphenyl)- | 0.3±0.01 | 0.12±0.01 | n.d. | n.d. |
|  | Methanone, (1-hydroxycyclohexyl)phenyl- | 0.14±0.01 | n.d. | 0.12±0 | 0.12±0 |
|  | Methyl Isobutyl Ketone | 0.66±0.09 | n.d. | n.d. | n.d. |
|  | Propanedioic acid, dihydroxy- | 0.36±0.31 | 0.16±0.01 | n.d. | n.d. |
| **Hydrocarbons** | 1,3-Cyclopentadiene, 1,3-bis(1-methylethyl)- | 0.3±0.04 | n.d. | n.d. | n.d. |
|  | 1,3,5-Heptatriene, (E,E)- | 0.32±0.1 | 0.3±0.04 | n.d. | n.d. |
|  | 1,3,7-Octatriene, 2,7-dimethyl- | n.d. | n.d. | 0.54±0.01 | 0.39±0.03 |
|  | 1,4-Cyclohexadiene, 1-methyl- | 0.35±0.09 | n.d. | n.d. | n.d. |
|  | 1,5,9-Undecatriene, 2,6,10-trimethyl-, (Z)- | 0.21±0.03 | n.d. | n.d. | n.d. |
|  | 1,7-Octadiene, 2-methyl-6-methylene- | 0.32±0.03 | 0.2±0.02 | 0.21±0.01 | n.d. |
|  | 1H-3a,7-Methanoazulene, 2,3,4,7,8,8a-hexahydro-3,6,8,8-tetramethyl-, [3R-(3a,3abeta,7beta,8aa)]- | 1.51±0.01 | n.d. | 0.25±0.02 | n.d. |
|  | 1H-3a,7-Methanoazulene, octahydro-3,8,8-trimethyl-6-methylene-, [3R-(3a,3abeta,7beta,8aa)]- | 0.17±0.02 | n.d. | n.d. | n.d. |
|  | 2,6-Dimethyl-1,3,5,7-octatetraene, E,E- | 1.5±0.06 | 0.92±0.06 | 1.04±0.06 | 0.58±0.09 |
|  | Bicyclo[2.2.1]hept-2-ene, 1,7,7-trimethyl- | 0.13±0 | 0.14±0.01 | 0.21±0.01 | n.d. |
|  | Bicyclo[2.2.1]heptane, 7,7-dimethyl-2-methylene- | 0.06±0 | n.d. | n.d. | n.d. |
|  | Cyclohexane, 1-ethenyl-1-methyl-2,4-bis(1-methylethenyl)- | 0.72±0.08 | n.d. | n.d. | n.d. |
|  | Cyclohexene, 1-ethyl-6-ethylidene- | 0.08±0.01 | n.d. | n.d. | n.d. |
|  | Cyclohexene, 3-(1,5-dimethyl-4-hexenyl)-6-methylene-, [S-(R*,S*)]- | 0.1±0.01 | n.d. | n.d. | n.d. |
|  | Cyclohexene, 4-[(1E)-1,5-dimethyl-1,4-hexadien-1-yl]-1-methyl- | n.d. | n.d. | n.d. | n.d. |
|  | Cyclohexene, 4-methyl-1-(1-methylethenyl)- | 0.11±0.01 | 0.08±0.01 | 0.09±0 | n.d. |
|  | Isomyocorene | 0.48±0.07 | 0.29±0.02 | 0.29±0 | 0.16±0.05 |
|  | Naphthalene, 1,2,3,4-tetrahydro-1,6-dimethyl-4-(1-methylethyl)-, (1S-cis)- | 2.05±0.25 | n.d. | 0.62±0.04 | n.d. |
|  | Propene | 1.38±0.13 | n.d. | n.d. | n.d. |
|  | Tetradecane | 5.73±1.39 | 3.38±0.45 | 3.94±0.06 | 3.13±0.36 |
|  | Tridecane, 5-methyl- | 0.36±0.04 | n.d. | n.d. | n.d. |
|  | Undecane, 2,8-dimethyl- | 0.27±0.04 | 0.16±0.05 | 0.2±0.02 | n.d. |
|  | Undecane, 4,7-dimethyl- | 0.1±0.01 | n.d. | 0.13±0 | n.d. |
| **Heterocyclic_Compounds** | 1,6-Dioxaspiro[4.4]nonane, 2-ethyl- | n.d. | n.d. | n.d. | 0.1±0 |
|  | 1H-Pyrazole, 4,5-dihydro-5-propyl- | 0.28±0.02 | 0.41±0.02 | n.d. | n.d. |
|  | 2-Pyrazoline, 1-isobutyl-3-methyl- | n.d. | n.d. | 0.36±0.02 | 0.29±0.01 |
|  | 2H-Benzotriazole, 2-ethyl- | 0.1±0 | 0.13±0.01 | 0.06±0 | 0.09±0.01 |
|  | 2H-Pyran, 3,6-dihydro-4-methyl-2-(2-methyl-1-propenyl)- | 0.06±0 | n.d. | n.d. | n.d. |
|  | Dibenzofuran | 0.06±0 | 0.04±0 | 0.04±0 | n.d. |
|  | Thiophene, 2-(1,1-dimethylethyl)- | n.d. | 0.09±0.03 | n.d. | n.d. |
| **Aldehydes** | 2-Hexenal, (E)- | 0.35±0.01 | n.d. | 0.87±0.04 | 1.11±0.09 |
|  | 2,6-Octadienal, 3,7-dimethyl-, (E)- | 0.2±0.06 | n.d. | n.d. | n.d. |
|  | 3-(4-Isopropylphenyl)-2-methylpropionaldehyde | n.d. | n.d. | n.d. | 0.09±0.01 |
|  | 3,5-di-tert-Butyl-4-hydroxybenzaldehyde | 0.53±0.06 | 0.39±0 | 0.39±0.03 | 0.44±0.02 |
|  | Benzaldehyde, 3-ethyl- | 0.06±0.01 | 0.05±0 | 0.08±0 | 0.21±0.01 |
|  | Benzoic acid, 2-formyl- | n.d. | n.d. | n.d. | 1.23±0.35 |
|  | Dihydroxyacetic acid | 0.16±0 | 0.14±0 | n.d. | n.d. |
|  | Dodecanal | 0.13±0.01 | n.d. | n.d. | n.d. |
|  | Nonanal | 0.84±0.19 | 0.89±0.31 | 0.65±0.04 | n.d. |
|  | Octanal | 0.11±0 | 0.09±0 | 0.09±0.07 | n.d. |
| **Esters** | 2-Butenoic acid, methyl ester | n.d. | n.d. | 2.16±0.13 | 0.23±0.01 |
|  | 2-Butenoic acid, methyl ester, (E)- | 0.51±0.09 | 5.62±0.19 | n.d. | n.d. |
|  | 2-Ethyl-n-butyric acid ethyl ester | 0.33±0.07 | n.d. | n.d. | n.d. |
|  | 2(3H)-Furanone, 5-ethenyldihydro-5-methyl- | 0.35±0.05 | n.d. | n.d. | n.d. |
|  | 2(3H)-Furanone, dihydro-5-(2-octenyl)-, (Z)- | 0.5±0.08 | n.d. | n.d. | n.d. |
|  | 2(3H)-Furanone, dihydro-5-methyl- | 0.06±0.01 | n.d. | n.d. | n.d. |
|  | 4-Octenoic acid, ethyl ester, (Z)- | 0.23±0.03 | 1.44±1.92 | n.d. | n.d. |
|  | 5-Oxotetrahydrofuran-2-carboxylic acid, ethyl ester | 0.39±0.04 | 0.99±0.06 | 0.64±0 | 1.07±0.04 |
|  | Benzoic acid, 2-methylpropyl ester | 0.53±0.09 | n.d. | n.d. | n.d. |
|  | Butanedioic acid, diethyl ester | 111.36±8.82 | 189.39±1.65 | 215.09±10.85 | 185.14±1.08 |
|  | Butanedioic acid, ethyl methyl ester | 0.9±0.14 | 1.89±0.02 | 2.36±0.07 | 1.4±0.03 |
|  | Butanoic acid, 2-methyl-, 1,2-dimethylpropyl ester | 0.41±0.47 | n.d. | n.d. | n.d. |
|  | Butanoic acid, 2-methyl-, 2-methylpropyl ester | 0.1±0 | 0.2±0.03 | 0.08±0 | n.d. |
|  | Butanoic acid, 2-methyl-, 3-methylbutyl ester | 0.3±0.01 | 0.29±0.02 | 0.21±0 | n.d. |
|  | Butanoic acid, 3-methyl-, 3-methylbutyl ester | 0.11±0.02 | n.d. | n.d. | n.d. |
|  | Butanoic acid, 3-methyl-, phenylmethyl ester | 0.22±0.05 | n.d. | n.d. | n.d. |
|  | Butanoic acid, 4-hydroxy- | 25.75±2.82 | n.d. | n.d. | n.d. |
|  | Butanoic acid, propyl ester | 0.18±0.01 | n.d. | n.d. | n.d. |
|  | d-Dodecalactone | 0.53±0.08 | 0.84±0.06 | 0.72±0.02 | 0.93±0.04 |
|  | Diethyl malonate | 0.28±0.04 | 1.55±0.01 | 1.32±0.16 | 1.76±0.05 |
|  | Diethyl Phthalate | 0±0 | n.d. | n.d. | n.d. |
|  | Dodecanoic acid, ethyl ester | 2.94±0.13 | 1.98±0.13 | 1.89±0.13 | 1.36±0.04 |
|  | Ethanol, 2-[2-(2-methoxyethoxy)ethoxy]-, acetate | n.d. | n.d. | 0.1±0.01 | 0.19±0.02 |
|  | Ethyl 2-hydroxy-3-phenylpropanoate | 2.93±0.41 | 5.24±0.11 | 4.56±0.39 | 7.08±0.18 |
|  | Ethyl 4-t-butylbenzoate | 0.3±0.01 | 0.3±0.04 | 0.3±0.02 | 0.25±0.09 |
|  | Ethyl 5-methylhexanoate | 38.2±0.11 | 0.1±0 | 1.12±0.04 | n.d. |
|  | Ethylene glycol di-n-butyrate | 2.45±0.28 | 2.76±0.02 | 0.83±0.02 | 0.34±0 |
|  | Ethylparaben | n.d. | 0.15±0.01 | n.d. | 0.11±0.01 |
|  | Heptanoic acid, ethyl ester | 1.92±0.22 | 1.31±0.21 | 1.87±0.06 | 0.62±0 |
|  | Hexanoic acid, propyl ester | 0.82±0.01 | 0.32±0.02 | 0.47±0.05 | n.d. |
|  | Methyl isovalerate | 0.4±0 | 0.12±0 | n.d. | n.d. |
|  | n-Propyl benzoate | 0.11±0.02 | n.d. | n.d. | n.d. |
|  | p-Toluic acid, 2-ethylhexyl ester | n.d. | 0.24±0.03 | 0.27±0.03 | 0.2±0.02 |
|  | Pentanedioic acid, 2-hydroxy, 1,5-diethyl ester | 0.11±0.01 | 0.53±0.01 | 0.23±0.03 | 0.37±0.03 |
|  | Pentanoic acid, 2-methyl-, methyl ester | 0.07±0.01 | n.d. | n.d. | n.d. |
|  | Pentanoic acid, 3-methyl-, ethyl ester | 0.06±0 | n.d. | n.d. | n.d. |
|  | Pentanoic acid, 4-oxo-, ethyl ester | 0.04±0.01 | 0.14±0 | n.d. | n.d. |
|  | Propanoic acid, 2-methyl-, 2-(hydroxymethyl)-1-propylbutyl ester | 0.05±0.01 | n.d. | n.d. | n.d. |
|  | Propanoic acid, 2-methyl-, 2-methylpropyl ester | 0.15±0.01 | 0.21±0.01 | n.d. | n.d. |
|  | Propanoic acid, 2,2-dimethyl-, butyl ester | 0.85±0.16 | n.d. | n.d. | n.d. |
|  | Undecanoic acid, ethyl ester | 0.47±0 | 0.29±0.03 | 0.3±0.03 | 0.14±0.01 |
| **Alcohols** | 2-methyl-1-Pentanol | n.d. | 0.22±0 | 0.42±0.02 | 0.16±0 |
|  | 1-Propanol | 24.38±3.05 | 22.88±0.15 | 24.8±0.99 | 19.49±0.65 |
|  | [2-Methyl-1-propanol](https://www.so.com/link?m=bRNCAvIW9tg5xxK7lElqskrSzdtLeN7qpSMsrU476VTYeofVAgLr+cMig0xWJusjRUqPiOsxosb6NLwfDBjkFBcXyEbBkvT0MemYTpVLoI+3H2BtnXm9gK5djIRPpX2241U0E3lW3iDoRngrxgAS8m/+7Pts590iuSRWgIsvP5VMKvxKIl8fPwr6mbLU=) | 196.21±226.5 | 95±4.04 | 335.74±15.98 | 324.61±9 |
|  | 1,6,10-Dodecatrien-3-ol, 3,7,11-trimethyl-, (E)- | 67.87±3.97 | 5.88±0.3 | n.d. | 6.16±8 |
|  | 11-Tridecyn-1-ol | 0.13±0.02 | n.d. | n.d. | n.d. |
|  | 2-Hexen-1-ol, (E)- | 0.32±0.05 | n.d. | 0.12±0.01 | n.d. |
|  | 4-methyl-2-Pentanol | 0.78±0.13 | 0.31±0.01 | n.d. | n.d. |
|  | 2,6,10-Dodecatrien-1-ol, 3,7,11-trimethyl-, (Z,E)- | 4.86±0.14 | n.d. | n.d. | n.d. |
|  | 3-Cyclohexene-1-ethanol, beta,4-dimethyl- | 1.05±0.17 | n.d. | 0.15±0 | n.d. |
|  | 3-Methyl-hepta-1,6-dien-3-ol | 0.09±0.01 | n.d. | n.d. | n.d. |
|  | 3-Octanol, 2,3-dimethyl- | 0.07±0 | n.d. | n.d. | n.d. |
|  | 3-Pentanol | n.d. | n.d. | n.d. | 0.12±0.01 |
|  | 4-Cyclopentene-1,3-diol, trans- | 0.47±0.05 | n.d. | n.d. | 6.15±2.03 |
|  | 4-Nonanol, 2,6,8-trimethyl- | 0.19±0.01 | n.d. | n.d. | n.d. |
|  | 5-Hepten-2-ol, 6-methyl- | 1.11±0.17 | 0.36±0.01 | 0.75±0.03 | 0.34±0 |
|  | 9-Dodecyn-1-ol | 0.08±0.01 | n.d. | n.d. | n.d. |
|  | Amylene hydrate | 0.07±0 | 0.1±0.04 | 0.08±0.02 | n.d. |
|  | Cyclopentanepropanol, 2-methylene- | 0.1±0 | n.d. | n.d. | n.d. |
|  | Eucalyptol | 0.25±0.02 | 0.32±0.01 | n.d. | n.d. |
|  | Isopropyl Alcohol | 5.14±1.57 | 11.36±0.04 | 5.69±0.07 | 2.27±0.96 |
|  | Linalool | 90.38±11.01 | 39.81±0.38 | 35.37±0.59 | 30.21±2.24 |
|  | p-Mentha-1,8-dien-7-ol | 0.19±0.03 | n.d. | n.d. | n.d. |
| **Carboxylic_Acids** | (R)-(-)-4-Methylhexanoic acid | 0.1±0.01 | 0.05±0 |  |  |
|  | 2-Hexenoic acid, (E)- | 1.6±0.27 | n.d. | n.d. | n.d. |
|  | Butanoic acid, 2-methyl- | 47.38±6.71 | 21.71±0.17 | 16.94±0.57 | n.d. |
|  | Heptanoic acid | 1.46±0.02 | 1.07±0.12 | 1.13±0.05 | 0.76±0.06 |
| **Others** | 1,4-Dihydronaphthalene | 0.73±0.22 | n.d. | n.d. | n.d. |
|  | 1,4-Dimethyl-2-cyclopentylbenzene | 0.07±0 | n.d. | 0.08±0.01 | n.d. |
|  | 2-Methylbutanoic anhydride | 0.07±0.01 | n.d. | n.d. | n.d. |
|  | 2,4-Diacetoxypentane | 0.23±0.01 | n.d. | n.d. | n.d. |
|  | 4-(2-(Acryloyloxy)ethoxy)-4-oxobutanoic acid | n.d. | 0.33±0.02 | n.d. | n.d. |
|  | 4-Acetyl-1-methylcyclohexene | 0.24±0.14 | n.d. | n.d. | n.d. |
|  | Azulene, 1,4-dimethyl-7-(1-methylethyl)- | 0.1±0.02 | n.d. | n.d. | n.d. |
|  | Benzene, (cyclopropylidenemethyl)- | n.d. | n.d. | 0.2±0.16 | 2.63±0.44 |
|  | Benzene, 1-ethenyl-4-methoxy- | 0.45±0.08 | 0.07±0 | 0.09±0 | n.d. |
|  | Benzene, 1-ethyl-2,4-dimethyl- | 0.11±0 | 0.14±0.01 | n.d. | n.d. |
|  | Benzene, 1,1'-(1,1,2,2-tetramethyl-1,2-ethanediyl)bis- | n.d. | 0.1±0.03 | n.d. | n.d. |
|  | Benzene, 1,1'-(1,2-cyclobutanediyl)bis-, cis- | 49.28±7.07 | n.d. | 45.89±6.77 | n.d. |
|  | Boronic acid, ethyl- | n.d. | n.d. | n.d. | 0.32±0.04 |
|  | Cyclopropylacetylene | n.d. | 1.41±0.57 | 1.13±0.09 | 1.15±0.12 |
|  | Dimethyl Sulfoxide | 0.09±0.01 | n.d. | 0.1±0 | n.d. |
|  | dl-Menthol | 0.34±0.01 | n.d. | n.d. | n.d. |
|  | Ethane, 1,1'-oxybis[2-methoxy- | n.d. | n.d. | 0.19±0.01 | 0.13±0.01 |
|  | Heptane, 2-(hexyloxy)- | 1.29±0.06 | n.d. | n.d. | n.d. |
|  | Isolongifolene, 4,5,9,10-dehydro- | 0.1±0 | n.d. | n.d. | n.d. |
|  | Naphthalene, 1,2,3,4-tetrahydro-1,1,6-trimethyl- | n.d. | 0.07±0.01 | 0.17±0 | 0.09±0.01 |
|  | Naphthalene, 1,6-dimethyl-4-(1-methylethyl)- | 0.28±0.04 | 0.03±0 | 0.12±0.01 | 0.06±0.03 |
|  | Naphthalene, 2-methyl-1-propyl- | 0.03±0 | n.d. | n.d. | n.d. |
|  | Naphthalene, 2,6-dimethyl- | n.d. | 0.12±0.01 | n.d. | n.d. |
|  | Phenol, 2,6-bis(1,1-dimethylethyl)-4-(1-methylpropyl)- | 0.19±0.03 | 0.14±0.01 | 0.16±0 | n.d. |
|  | 1-Propanol, 3-(methylthio)- | n.d. | n.d. | 0.32±0.02 | n.d. |
|  | 1,3-Dioxolane, 2-methoxymethyl-2,4,5-trimethyl- | 0.18±0.03 | 0.3±0.01 | 0.15±0 | n.d. |

**Note:** n.d. Indicates that the volatile component is not detected.
