# Supplementary material for: Comparison of Flavor Differences between the Juices and Wines of Four Strawberry Cultivars Using Two-Dimensional Gas Chromatography-Time-of-Flight Mass Spectrometry and Sensory Evaluation
Source: Molecules. 2024 Oct 3;29(19):4691. doi: 10.3390/molecules29194691 (PMC11477698; doi:10.3390/molecules29194691)
Supplement: Supplementary file 1 [file molecules-29-04691-s001.zip › Table S3. ROAV values of different VOCs in samples.doc]

**Table S3.** ROAV values of different VOCs in samples

| VOCs | | Odor Character | ROAV values of different samples | | | | | | | |
| --- | --- | --- | --- | --- | --- | --- | --- | --- | --- | --- |
|  |  |  | BX | TCL | TZ | ZJ | BX_J | TCL_J | TZ_J | ZJ_J |
| 1-Octen-3-ol | Alcohols | Grassy, Fruity, Dusty, Oily | 1.13 | 0.83 | 1.81 | 2.61 | 4.56 | 88.07 | 5.34 | 89.13 |
| Benzyl alcohol |  | Floral, Fruity, Sweet, Estery | 0 | 0 | 0 | 1.03 | 0 | 0 | 0 | 0 |
| 2-Heptanol |  | Peach Fragrance, Fusel Oil Smell, Fruit Fragrance, Floral Fragrance, Honey Fragrance | 0.05 | 0.02 | 0.03 | 0.01 | 0.08 | 0.82 | 0.16 | 1.01 |
| 1-Butanol |  | Fruity | 0.03 | 0.04 | 0.04 | 0.04 | 0 | 0.05 | 0 | 0.01 |
| Octanal | Aldehydes | Grassy, Fruity | 0.01 | 0 | 0 | 0 | 0.15 | 0.12 | 0.11 | 2.29 |
| Nonanal |  | Soap, Grass, Water Smell | 0.04 | 0.03 | 0.02 | 0.03 | 0.13 | 0 | 0 | 1.59 |
| Hexanal |  | Floral, Fruity | 0 | 0 | 0 | 0 | 2.52 | 0 | 0 | 7.65 |
| Butanal, 3-methyl- |  | Floral, Fruity | 0.76 | 1.7 | 0.81 | 1.54 | 0.23 | 5.23 | 0.82 | 19.24 |
| butanoic acid 3-methyl- ethyl ester | Esters | Apple Fragrance, Pineapple Fragrance, Banana Fragrance, Fruit Fragrance | 10.63 | 3.46 | 1.49 | 2.71 | 100 | 22.8 | 5.17 | 100 |
| Propanoic acid, ethyl ester |  | Banana, Fruity | 0 | 0 | 0 | 0 | 0 | 0.06 | 0 | 0.03 |
| Ethyl Acetate |  | Pineapple, Apple, Fruit | 0.05 | 0.09 | 0.05 | 0.01 | 0.01 | 0.05 | 0.01 | 0.36 |
| Propanoic acid, 2-methyl-, ethyl ester |  | Sweet-Scented Osmanthus, Apple, Peach, Fruit | 2.99 | 5.14 | 2 | 3.19 | 1.19 | 0.64 | 0.13 | 3.07 |
| 3-Methyl-1-butanol |  | Banana, Sweet, Apple, Fruit Candy | 12.36 | 10.03 | 6.95 | 13.61 | 1.55 | 20.06 | 4.63 | 2.89 |
| Pentanoic acid, ethyl ester |  | Peach, Fruity, Floral, Sweet | 0.29 | 0.18 | 0.15 | 0.22 | 0.26 | 1.07 | 0.06 | 1.21 |
| Propanoic acid, 2-hydroxy-, ethyl ester |  | Sweet, Fruity, Grassy | 0 | 0 | 0 | 0 | 0 | 1.72 | 0 | 2.36 |
| Nonanoic acid, ethyl ester |  | Ester, Honey, Fruit | 0.01 | 0 | 0.02 | 0 | 0 | 0.02 | 0.01 | 0 |
| Hexanoic acid ethyl ester |  | Sweet, Fruity, Savory, Cucumber | 22.52 | 32.76 | 22.61 | 32.41 | 12.84 | 99.19 | 19.09 | 87.5 |
| Hexanoic acid, propyl ester |  | Fruity, Ester, Pineapple, Sweet | 0 | 0 | 0 | 0 | 0 | 1.73 | 0 | 2.36 |
| Butanedioic acid, diethyl ester |  | Fruity, Floral, Pollen | 0 | 0 | 0 | 0 | 1.39 | 6.25 | 0 | 8.55 |
| Decanoic acid, ethyl ester |  | Pineapple, Fruity, Floral | 0.05 | 0.04 | 0.05 | 0.05 | 0 | 0.32 | 0.51 | 0.05 |
| Butanoic acid ethyl ester |  | Apple, Pineapple, Fruity, Floral | 6.24 | 5.4 | 3.94 | 3.55 | 9.61 | 58.11 | 5.68 | 93.93 |
| Benzenepropanoic acid, ethyl ester |  | Honey Pineapple Fragrance, Fruit Candy Fragrance, Honey Fragrance, Fruit Fragrance, Floral Fragrance | 6.95 | 0.05 | 1.38 | 0.17 | 0.09 | 0.09 | 0.09 | 0.02 |
| 1-Propanol, 3-(methylthio)- | Ethers | Glue Smell, Boiled Carrot Smell, Rubber Smell | 0 | 0 | 0 | 0 | 8.81 | 0.01 | 0 | 1.43 |
| n-Decanoic acid | Acid | Goat Stink, Wine Stink, Rubber Stink, Paint Stink, Animal Stink | 0 | 0 | 0 | 0 | 0 | 0.05 | 0.04 | 0.02 |
| Octanoic acid |  | Fruity, Floral, Oily | 0.11 | 0.14 | 0.14 | 0.18 | 0.03 | 0.53 | 0.49 | 0.51 |
| Nonanoic acid |  | Fat Smell | 0.02 | 0.02 | 0.01 | 0.02 | 0 | 0.14 | 0.02 | 0.12 |
| Hexanoic acid |  | Sweat Smell, Animal Smell, Sour Smell, Sweet Smell, Fruit Smell | 0.28 | 0.19 | 0.15 | 0.2 | 0.24 | 1.68 | 0.26 | 1.85 |
| Butanoic acid |  | Sweat Odor, Sour Odor, Pit Mud Odor | 0 | 0.05 | 0.02 | 0.03 | 0.06 | 2.28 | 0.01 | 3.13 |
| Butanoic acid, 2-methyl- |  | Sweat Odor, Sour Odor, Pit Mud Odor | 0.037 | 0.02 | 0.01 | 7.11 | 0.05 | 0.32 | 0.01 | 0.15 |
| Pyrazine, 2,5-dimethyl- | Organoheterocyclic compounds | Green Grass, Fried Bean Spice | 0 | 0 | 0 | 0 | 5.81 | 0 | 9.96 | 9.43 |
| Octanoic acid ethyl ester |  | Pear Fragrance, Lychee Fragrance, Fruit Fragrance, Sweet Fragrance, Lily Fragrance | 100 | 100 | 100 | 100 | 0.5 | 22.06 | 100 | 4.48 |
| Dimethyl disulfide | Organo sulfur compounds | Glue Smell, Boiled Carrot Smell, Rubber Smell | 0 | 0.05 | 0.01 | 0.02 | 0.07 | 0.63 | 0 | 0.68 |
